# What Is eHealth (6): Perspectives on the Evolution of eHealth Research

**DOI:** 10.2196/jmir.8.1.e4

**Published:** 2006-03-31

**Authors:** David K Ahern, Jennifer M Kreslake, Judith M Phalen

**Affiliations:** ^1^Health e-Technologies InitiativeBrigham and Women’s HospitalBostonMAUSA

**Keywords:** Health services research, outcome and process assessments (health care), behavioral medicine, health behavior, information dissemination, telemedicine

## Abstract

**Background:**

The field of eHealth holds promise for supporting and enabling health behavior change and the prevention and management of chronic disease.

**Objective:**

In order to establish areas of congruence and controversy among contributors to the early development, evaluation, and dissemination of eHealth applications, as well as the desire to inform an evaluation research funding agenda, 38 semistructured, qualitative interviews were conducted among stakeholders in eHealth between May 2002 and September 2003.

**Methods:**

Participants were asked about their perspectives on the credibility, value, and future potential of information technology for health behavior change and chronic disease management. Interviews were coded and analyzed for emergent themes using qualitative methods.

**Results:**

Consistent themes were identified across stakeholder groups, with slight differences in emphasis. These topics included the following: (1) consensus and standardization—most stakeholders expressed a strong desire for a more coordinated, rigorous effort to define and integrate the field; (2) evaluation methods and challenges—demonstrating outcomes is required to establish eHealth quality and efficacy, but stakeholders were not satisfied with the sensitivity, validity, and reliability of existing outcome measures; (3) quality, value, and future potential—the intersection between eHealth’s potential cost-effectiveness, efficiency, and improved clinical status among users generated a high degree of interest; and (4) health disparities—many stakeholders contended that traditionally underserved populations will particularly benefit from eHealth applications, although others argued that the underserved are also disadvantaged in terms of access to technology.

**Conclusions:**

Recommendations included the need for improvement and formalization of development and evaluation standards across private and public sectors, additional research on the technology needs and preferences of traditionally underserved populations, and long-term epidemiologic studies of the impact of eHealth on outcomes and cost-effectiveness.

## Introduction

The importance of chronic disease prevention and management becomes clear when the combined effects of the projected aging of the US population, the limited capacity of the existing health care system to support the increasing demands of an older population, and the continued rise in health care spending are considered [1–5]. An emerging approach for reducing the burden of chronic disease involves engaging patients and consumers in health promotion activities (e.g., healthy eating and increased physical activity), which require sustained behavior change. Research has noted the significant role that prevention can play in reducing morbidity and mortality [6–9], and addressing risk behaviors can be an efficient way to prevent or manage chronic illness in populations. However, even in high-quality health care settings, counseling and monitoring are time consuming and often impossible for clinicians to do in a rigorous, consistent way with all of their at-risk patients [10–13]. It is also important to note that traditionally underserved populations are the most likely to have multiple behavioral risk factors, and the least likely to have access to consistent, quality care [14,15].

eHealth is emerging as a promising vehicle to address the limited capacity of the health care system to provide health behavior change and chronic disease management interventions. For the purposes of this paper, eHealth is defined as the use of emerging interactive technologies (e.g., Internet, CD-ROMs, personal digital assistants, interactive television and voice response systems, computer kiosks, and mobile computing) to enable health improvement and health care services [16]. Though still at an early stage of development, the evidence base is growing for these types of technology-based interventions. eHealth programs offer the potential for enhanced reach, including traditionally underserved populations, at relatively low cost; scalability; time efficiency; and the capacity to provide tailoring and customization for individual patients and consumers. Despite these potential benefits, there are barriers to the full implementation of eHealth solutions, and the limitations of access, health and technology literacy, and quality measures must be addressed [17,18].

While no single entity or sector originated the idea of harnessing electronic communication technology to address health care issues, purchasers (e.g., health management organizations), physicians, other practitioners, health care delivery systems, patients (referred to in this paper as users), developers, and academics all bring unique perspectives to, and have sometimes divergent opinions about, maximizing eHealth’s potential. In the realm of health behavior change and disease management, there had been an increasing call to explore research methodologies for eHealth evaluation research, how these technologies could be created and adapted to reach traditionally underserved populations, and the formation and implementation of standards for the assessment of interventions [19].

In 2002, The Robert Wood Johnson Foundation created the Health e-Technologies Initiative, a national program office focused on expanding the body of knowledge about the efficacy, cost-effectiveness, and overall quality of eHealth applications for health behavior change and chronic disease management. To establish a cohesive set of funding priorities, it was necessary for the Health e-Technologies Initiative to consider perspectives from a broad range of sectors, comparing areas of overlap and addressing controversies. A series of interviews was conducted among opinion leaders (stakeholders) in eHealth in order to assess the existing strengths and challenges in eHealth evaluation research for health behavior change and chronic disease management. Qualitative inquiry provided an opportunity for individuals who represented varied interests to “make their case,” which, when aggregated with the perspectives of others, revealed a previously undocumented state of the field.

## Methods

From May 2002 to September 2003, 38 qualitative interviews were conducted. Each discussion consisted of two interviewers and between one and five participants. Participants were recruited by convenience sampling from designated sectors involved in the development, evaluation, dissemination, or use of eHealth technologies. Specifically, the recruited sample consisted of individuals in the following categories: established developers/researchers of interactive health communications (IHCs); opinion leaders in information technology; projects/programs that have implemented IHCs; health plan representatives; technology and health care futurists; physician organizations/provider groups; purchasers (public/private coalitions)/larger employers; consumer groups; data collectors; and pharmaceuticals. Participants were distributed throughout the United States, with 18 in the Northeastern region (Massachusetts, Rhode Island, New York), 7 in the Mid-Atlantic region (Washington, DC; Pennsylvania), 5 in the Midwest (Wisconsin, Idaho, Minnesota, Michigan, and Illinois), 6 in Western states (Oregon, California, Arizona), and 2 in the South (North Carolina and Missouri). Interviews were conducted in person whenever possible, but due to geographic limitations, one third of interviews were conducted by telephone.

The unit of analysis for this study was each interview session, rather than the individual respondents. A total of 9 interviews were conducted with developers and researchers, 7 with opinion leaders in information technology, 4 with projects and programs that use IHCs, 4 with health plan representatives, 4 with technology and health care futurists, 3 with physician organizations and provider groups, 2 with purchasers and larger employers, consumer groups, and data collectors, and 1 with a pharmaceutical company.

Participants consented to be audiotape recorded and received copies of their transcribed interviews to modify or edit, as necessary. Interviews lasted approximately 50 minutes. Participants were informed that their individual responses would remain confidential but would be aggregated for future qualitative data analysis and that quotes would not be attributed to individuals unless explicit written consent was obtained prior to doing so.

The following questions were asked:

- What is your current view of the credibility, quality, and validity of eHealth technology (defined how) for health behavior change and chronic disease management? In general, do you believe it is effective? Why or why not?

- Could you provide any examples of current eHealth programs for health behavior change and chronic disease management that you believe to be effective? How were these developed? How do these work? How do you know they're effective? How are they evaluated?

- How would you go about evaluating the cost-effectiveness and quality (defined how) of eHealth programs for health behavior change and chronic disease management? What measurements would you use (quality adjusted life years, economic metrics, satisfaction, other health outcomes, etc.)?

- What type of experimental methods would you use to evaluate programs for health behavior change and chronic disease management (controlled studies, quasi-experimental studies, natural experiments, modeling, etc.)? Is there a gold standard? How is it achieved?

- What obstacles exist to effectively evaluating eHealth for health behavior change and chronic disease management? What could be done to overcome these?

- What are your perceptions about traditionally underserved populations and eHealth access? How do you define access (hardware, primary speaking/reading language, reading levels, basic knowledge of technical/computer training, etc.)? How can access be strengthened for these populations?

If the participants asked for a definition of eHealth, they were encouraged to offer their own definition, and their comments were not restricted solely to IHCs. A spectrum of individual, community, and health care applications were discussed according to sector, but the line of inquiry focused primarily on issues of quality in the development and evaluation of IHCs geared toward health behavior change or chronic disease management due to the nature of the questions being asked.

Transcripts were read line-by-line and coded for primary categories using NVIVO qualitative analysis software (version 2.0, QSR International)*.* Frequent or related categories were grouped and identified as second- or third-level codes. When necessary, tape-recorded interviews were revisited for clarification of the participant’s tone and intended meaning. As relationships between codes became evident, themes began to emerge. Table 1 provides an overview of the relative emphasis of topic area by stakeholder category.

**Table 1:** Areas of emphasis by stakeholder group (✓ means prominently addressed by the majority of interviews within indicated sector; -- means minimally addressed or not at all)*

	**Developers/ Researchers**	**Health Plan Representatives**	**Programs and Projects Using IHCs**	**Opinion Leaders of Technology in Health Care**	**Futurists**	**Physician Organizations/Provider Groups**
Access to technology (health disparities)	✓	✓	✓	✓		
Cost-effectiveness	✓	✓		✓		
Process measures	✓				✓	✓
Outcome measures	✓	✓		✓		
Utility (eHealth quality and value)	✓	✓		✓		
Funding for evaluation (obstacle to evaluation)		✓	✓			
Market pressures (eHealth quality and value)		✓	✓			
Infrastructure				✓		✓
Utilization rates and patterns	✓	✓				
Credibility among opinion leaders			✓			✓
Funding for dissemination (obstacle to dissemination)			✓			✓
Reimbursement incentives				✓		✓
						
Translation from research to practice	--	--	--		--	--
Patient-provider tension	--	--		--	--	--
Privacy concerns	--	--	--	--		
Reliability (evaluation approaches)	--	--		--		--
Generalizability		--	--			--
Credibility among providers	--		--			--
Liability	--	--		--		
Consistency of care		--	--		--	
Combined with standard care		--	--	--		

^*^Data collectors, purchasers, pharmaceuticals, and consumer group representatives excluded because of small sample size (≤ 2 interviews)

## Results

### Theme 1: Consensus and Standardization

There was universal frustration with the lack of comparability and standardization within the domain of eHealth. Stakeholders expressed a strong desire for a coordinated, rigorous effort to define and integrate the field. Researchers, as well as purchasers, need criteria for identifying quality information, sharing and comparing findings, and building upon current evidence in order to move eHealth forward. According to one stakeholder, “The most critical [challenge] is not working in isolation and certainly understanding what other people are doing in this arena. We…don’t want to reinvent the wheel.”

The dearth of consensus and standardization in development and evaluation activities often appeared implicitly in stakeholder discussions of other topics and themes cited throughout this paper; many of the challenges identified by stakeholders pointed toward the larger incongruities surrounding the field of eHealth. In order to standardize measures and ensure comparable results, an overarching paradigm must be well defined. Stakeholders were troubled by the broad, amorphous definitions of *eHealth* and *behavior modification.* At the time the interviews were conducted, professional organizations such as the Disease Management Association of America were beginning to issue guidelines and recommendations for determining the value of these interventions [20,21], and these efforts were highly valued by the researchers in this sample. More recent publications have continued to address the varying meanings of the word *eHealth* [22–26].

### Theme 2: Evaluation Methods and Challenges

The stakeholders explained the relative importance, from their perspective, of refining process and outcome measures, determining the optimal study designs to capture these factors, and the relevance of the eHealth research environment to interactive applications already being disseminated in health care and commercial industry. Randomized controlled trials were regarded as the “gold standard” for evaluating application effectiveness, but stakeholders noted that eHealth presented unique challenges to this study design. These results align very closely with the issues raised in an editorial in this journal that was published shortly before the interviews were conducted. It is difficult to determine the degree to which this article, and any surrounding discussions in the literature, influenced the responses, particularly since no interviews occurred prior to its publication [27].

### Process Measures

The stakeholders discussed the challenges associated with measuring usage, particularly traffic and utilization, using quantitative and qualitative methods. Process measures provide insight into influences on utilization and can explain associations between differential attrition and outcome status [28]. Identifying and accurately measuring variances within the length of delay that users experience when trying to access the Internet, the time a user spends on a page, which components of the program are used more than others, and the validity of responses to online questionnaires were examples of process measures cited by the stakeholders. Additionally, stakeholders were concerned with more qualitative measures, such as how the user interpreted the information that was presented, the degree of comprehension, and the user’s level of engagement in the program. There was a concern expressed among stakeholders that if the delivery mechanisms are not well understood and validated, the outcome results will be difficult to interpret. Without process refinement, randomized controlled trial results may not be accurate and could threaten the credibility, perceived effectiveness, and, ultimately, the uptake of these technologies. Only researchers and developers commented on process measures in any level of detail and were mainly concerned that, from their perspective, quality design was not emphasized by funders and purchasers. “It’s not, ‘if you build it, they will come,’” noted one developer. Process measures help those designing interventions understand user interests and learning styles, which greatly impacts the program uptake and effectiveness. Users who are actively engaged in eHealth applications may benefit more than those who interact in a superficial way with the program. Developers and researchers expressed an interest in the education literature, particularly its research on methods of learning, in guiding the creation of applications that are appealing and relevant to users. Collaborations between educational researchers and eHealth developers may facilitate the construction of well-designed, effective instructional programs that can adapt to individual styles of learning.

A major criticism of current data collection methods was that they do not distinguish among usage behaviors. For example, if tracking reveals that a Web page is viewed for an extended period of time, it does not tell evaluators how long a user is interacting with the page, or if the user is even sitting at the computer. Furthermore, it is difficult to correlate navigation patterns with users’ cognitive factors related to behavior change, such as comprehension or interest in content. Commonly used measures (including hits, time on page, number of log-ins) all have disadvantages, and at the time of the interviews, no ideal measure or measures of usage had emerged as an optimal industry standard. While there was a sense of dissatisfaction with process measures, they were viewed as fundamentally important to building an effective intervention, and their role in development and evaluation should be as highly regarded as outcome measures.

### Outcome Measures

Ultimately, the credibility and value of eHealth lies in its ability to demonstrate positive outcome effects. It was universally understood that funders and purchasers expect proof that an intervention is effective, although there was uncertainty as to what level of rigor was sufficient. It is difficult to determine quality outcome measures, especially when constrained by short follow-up periods. In lieu of long-term clinical outcomes (which require follow-up years later, and few studies have been performed on eHealth applications) or population-level measures of impact (i.e., a significant reduction in disease that can be attributed directly to an eHealth intervention, or a rigorous cost-effectiveness analysis), demonstrated behavior change was considered to be a good proxy measure and was considered a more robust indicator of intervention success than reported improvements in knowledge and comprehension.

Evaluating behavioral components addressed by IHCs was considered to be a major challenge. Instruments that have been validated to measure behavior change have often not been validated for the evaluation of online interventions and therefore were considered too general. Qualitative, self-report, and Likert scales were named as helpful in obtaining certain types of information, but objective evidence of behavior change was preferred over self-reported measures or patient satisfaction ratings. As one participant pointed out, “Just because someone likes an intervention doesn’t mean it’s doing them any good.” That being said, user satisfaction is not irrelevant to the efficacy of an intervention because satisfaction with a program may influence utilization, which may impact eventual clinical outcomes.

The extent to which process and outcome are intertwined was a consistent theme among developers, researchers, and IT opinion leaders, but was also recognized by the other stakeholders as well. Patient, health plan, and physician representatives were particularly conscious of the importance of user satisfaction, which may reflect the proximity of these stakeholders to patients and their perceived quality of care from their doctors and health insurers.

### Study Design

Time and expense were the most consistently, emphatically cited challenges to rigorous evaluation. Researchers and developers were particularly frustrated with the separateness of funding streams for development and evaluation activities. Stakeholders involved in research and development regarded the creation of an intervention and its evaluation to be a cyclical process; evaluation findings provide valuable feedback to designers of the intervention, but funders’ priorities and limitations mandate the process to be more linear in nature. While accepting of the tension that often exists between what they want to discover and their obligations to fit within the parameters of a grant, researchers and developers find it more challenging to reconcile the choice they often face between allocating limited resources (time, money, personnel) to either development or evaluation. When required to choose, development is favored, with the rationale that it is pointless to evaluate poorly constructed interventions.

There are caveats to setting the minimum bar at the level of randomized controlled trials. If this design is considered to be the only acceptable methodology, there was concern that the rate of research will be too slow to keep up with development. “The research paradigm doesn’t match the context,” according to one stakeholder, and it was recommended that before attempting an randomized controlled trial, it is important to make certain that the technology and process measures are proven, even at a “lower level” than a randomized controlled trial. Without process refinement, randomized trial results may not be accurate, and stakeholders were concerned that questionable results may threaten the credibility of eHealth:

eHealth Researcher We can throw a lot of money at randomized trials that are the first thing out of the gate, and [a good number] of them will come up negative. And the whole idea of eHealth will be besmirched and perhaps inappropriately abandoned because we went into it too fast.

Alternative, potentially more practical methods include usability and case-control designs, which more easily align with implementation timelines. eHealth applications present unique methodological challenges, which are outlined in Table 2.

**Table 2:** Methodological concerns in eHealth evaluation

**Selection Bias**	Recruiting representative populations of interest is limited by users’ access and technological literacy.
**Confounding and Effect Measure Modification**	Controlling for unknown confounders (baseline severity of condition, comorbidity) is especially difficult when evaluating discrete eHealth interventions; quasi-experimental designs, case-control studies, and field trials may not accurately measure impact.
**History**	Health care and technology are in a constant state of rapid change, which may change participants’ experiences during the course of a trial or evaluation.
**Attrition**	If a large proportion of participants in the intervention group stop using the application, statistical power is reduced and results are biased toward the null. Differential attrition can occur across condition or across level of technological proficiency.
**Contamination**	As eHealth programs become more ubiquitous, it will be challenging to find an unexposed control population.

Stakeholders were unable to propose solutions to major sampling challenges associated with Internet research:

eHealth Developer By far and away, the biggest challenge for doing our kind of work on the Internet is to be able to get the kind of proactive recruitment rates that we’ve been able to do using other technologies. Talking with researchers, I know that that’s one of the major challenges to get adequate percentages of people participating.

As with the development of mail and telephone surveys in previous decades, online surveys and recruitment strategies need to be validated. It was difficult for researchers to define a general online population, and several felt that, while some response rates in eHealth research can appear to be strong, they were uncomfortable assuming these respondents were at all representative of the populations of interest (see “selection bias” in Table 3]. For example, they highlighted the need to prevent multiple responses from a single user through internal filtering mechanisms, particularly when incentives were offered to survey participants. Determining the size and key characteristics of the sample population was not just of concern to researchers; one health plan purchaser noted that he/she “liked to see denominators and see what percentage of eligibles have been using this in a given time period.” However, methods for accurately measuring these factors were not yet well developed at the time of the interviews.

The increasing presence of the Internet in the daily lives of individuals [29] may make it increasingly difficult to recruit controls who do not have some baseline exposure to similar eHealth programs, and to prevent contamination. If eHealth applications that resemble the one being evaluated exist elsewhere, there is a possibility that users may access these interventions independently, potentially receiving some “dose” of a similar intervention (particularly those that concern specific conditions or behaviors). Stakeholders commented on the importance of assessing research participants’ exposure to other eHealth applications at baseline and follow-up. Additionally, due to the stratification of information technology access along socioeconomic lines, evaluation results of eHealth applications may be particularly prone to bias if the sample does not accurately represent the target population:

IT Opinion Leader There’s a feeling that the patients who are most likely to make use of Internet-based health care tools are the least likely, in many cases, to need it. So if you’re trying to show an effect in terms of chronic disease getting much better, the people who today have access to the tools and would use them are already doing fairly well. The chance of showing the effect is smaller because of that baseline being higher than the folks who are unconnected, don’t have access, [are] doing poorly, but possibly also lacking the motivation or the resources or the connections in various ways to give them access.

Those whom eHealth applications may benefit most must be represented in sample selection. Stakeholders contended that these individuals might be those who have little or no access to other sources of care. If a sample is not representative of these users, but instead is made up of participants who, overall, have higher access (to health care, to eHealth tools, to healthy lifestyle choices and preventive care) due to higher socioeconomic status, researchers may encounter problems in demonstrating the effects of eHealth applications. Therefore, it is crucial that sampling methods continue to be refined and validated in order to accurately determine the efficacy of eHealth in the populations it has the potential to reach.

### Perceived Credibility Among Purchasers and Users

Randomized controlled trials can limit an application’s time to market, and interest in dissemination should be balanced with the level of rigor expected of an evaluation. Creators of interventions felt intense pressure to develop products that are efficacious and usable from the beginning and are palatable to the public and physicians. However, stakeholders were aware that end users and some purchasers are not necessarily as concerned with evidence-based proof of effectiveness. As an IT opinion leader noted with chagrin, “The things that tend to lead people to trust a system are not the kinds of things that probably indicate the quality of a system…. People tend to believe in stuff that’s flashy, rather than in-depth.”

### Theme 3: Quality, Value, and Future Potential

All stakeholders were concerned about the dearth of quality control or regulatory entities concerning eHealth, and many recommended a rating system to distinguish legitimate online sites from ones that are merely attractive or popular. “[I]t’s like the Wild West out there,” said a physician. “There are selected good resources. Connecting patients with the right resources is a huge challenge.” The stakeholders eschewed a free-market mentality when it came to users choosing IHCs as they would any other consumer product. As a component of health care, it was unanimously held that these applications should be tested and ranked in terms of quality in a similar fashion as other treatment regimens. The controversy concerned the identification of methodologies that are necessary and realistic to reconcile the demands of good science and consumer interest. Even with these concerns, the low cost, wide reach, potential for targeting audiences and tailoring to individuals, and interactivity of eHealth drove optimism for its future potential.

### Information Acquisition and Continuity of Care

During its brief history, eHealth has often been used for different purposes by physicians and patients. Patients were using eHealth, especially the Internet, in order to obtain more health information than they typically had access to within their patient-physician relationship.

This enhanced information acquisition began to trigger a shift in the role of the patient and physician, the impact of which has yet to be fully established. Ideally, though, eHealth may empower the patient to more actively participate as a member of the health care team, but stakeholders believed that physicians are key to realizing this objective. Stakeholders noted that physicians might be concerned over the quality of information obtained by patients online and are uncertain about whether the patient-driven inquisitiveness it generates will result in burdensome workloads. In a potentially cyclical and ironic pattern, reluctance by physicians to respond to patients’ inquiries may fuel greater interest in online information sources; such reliance would further underscore the need for vetted online health information.

While the computer does not replace the physician, it can function as an aid, helping physicians gain a deeper understanding of current best practices in the context of individual patients. Optimally, health care should be continuous, and technology’s ability to bridge time and geography makes it well suited to a longitudinal approach to care. The coordination of care providers (be they lay or professional) requires teamwork and both synchronous and asynchronous communication, and physician group members and eHealth researchers alike recognized this as the ideal approach to chronic disease management. By empowering the patient and enhancing patient-physician (and physician-physician) communication, eHealth may enable a shift from the traditional model of the physician -patient relationship to what one stakeholder referred to as a “patient–health care interface,” where patients would move beyond simple information collection to becoming fully integrated members of their care team by improving, for example, disease self-management strategies.

While stakeholders could easily envision several dimensions in which health care and patients, particularly for behavior change and chronic disease management, could benefit from opportunities for rapid information exchange offered by eHealth applications, “no one holds the whole story,” said one participant, “and I don’t think that I have seen any situations in which [technology] is captured in any significant way that can really help providers make decisions, and to learn from one another on how they are approaching it, and come to a consorted plan that is really in alignment with what the patient’s goals are.” It is clear that more research is needed on the appropriate and efficacious use of technology in efforts to integrate care.

In addition to eHealth’s role in clinical settings, stakeholders contended that patients are inevitably going to use the Internet for a variety of activities, and participants recognized the value in capitalizing upon the existing interest and skill sets to bring relevant health information to patients. As one behavioral psychologist pointed out, most people seeking health information are in the early stages of behavior change (pre-contemplation or contemplation). eHealth is a less costly way of engaging them and “holding their hand” into the later stages and optimizing behavioral results.

### Tailored eHealth Interventions

eHealth can differ from traditional, paper-based educational materials because of its ability to be customized according to user characteristics. While this was assumed by many stakeholders to have a greater potential to engage and encourage individuals toward behavior change than general information, this theory was somewhat controversial and stakeholders were not completely satisfied with the preliminary research that had been done comparing the efficacy of generic patient education with highly personalized or “tailored” materials. Additionally, achieving robust, comparable samples when measuring the efficacy of tailored interventions is challenging. Methodological concerns arise when evaluating the effectiveness of tailored messaging programs because, by definition, participants are not actually receiving exactly the same intervention. More advanced analysis strategies need to be applied in order to adequately accommodate this issue. 

Tailoring requires considerable design and development work, and some stakeholders discussed the possibility that money spent on tailoring should, instead, be concentrated on widespread dissemination of untailored messages to achieve maximum population-level impact. In order to combine the presumed efficacy of tailoring with the desired reach of eHealth, it was suggested that tailored information also be applied to populations with similar characteristics, allowing interventions to effectively target high-risk groups. Targeting populations at greater risk of chronic disease is thought to result in long-term savings to the health care system. After an initial investment in development, eHealth has a relatively lower delivery cost than traditional methods and has significant potential if incorporated into the existing health care infrastructure.

### Cost-Effectiveness

From a public health perspective, a return on investment was viewed as probable, since, according to one IT opinion leader, “The cost of providing these services is much lower than the alternatives. Much lower. Even if it’s less effective, and I’m not sure it is less effective yet.” Analyses on cost-effectiveness were strongly urged, although stakeholders acknowledged that cost measures are extremely difficult to determine. Any approach to cost-effectiveness was seen as dependent upon proven positive outcomes; eHealth’s value is contingent upon it being lower in cost than standard care as well as being empirically proven to be effective.

### Obstacles to Dissemination

The impact of eHealth on behavior change will be mitigated by environmental and infrastructure challenges, and this was acknowledged by all stakeholders. eHealth was not regarded as a “fix for the broken health care system” by any stakeholders; at most, said one stakeholder, the Internet can be a tool to help *systems* handle health care, but “it is not something that is going to fix health care all by itself.”

eHealth’s potential to maximize physicians’ limited time and contact with patients was highly regarded, although some were pessimistic about the degree to which physicians would embrace these technologies. For health care professionals, significant barriers to adoption exist at the point of care, including the financial costs of purchasing and installing systems, the disruption to office workflow, and the current lack of reimbursement for interacting with patients electronically via secure messaging/email or Web visits. Unless these issues can be resolved, adoption of eHealth solutions by physicians will be impacted negatively.

Interoperability is also an important aspect of eHealth. While researchers and developers were concerned with standardization of evaluation methodologies, stakeholders involved in the delivery of eHealth programs within health care identified an urgent need for standardized platforms to facilitate widespread use of the technology. As a leader of a program that has implemented IHCs, one stakeholder urged organizations to “move away from proprietary pieces, and work closer together for the greater good, and in some cases that means moving towards standards…simply to be able to get beyond the hurdle of technology or implementation or detail aspects, and get to the real goal which is stronger outcomes, better solutions, easier analysis.” It was believed that regulatory entities, as mentioned elsewhere in this article, could additionally help to manage inefficient connectivity and proprietary interests that prevent the effective interoperability of eHealth programs.

While eHealth was not purported to be the answer for all of the health care system’s woes, demographic shifts will eventually overload exclusively human-intensive interventions, since these systems are already understaffed and suffering from limited resources. “So it’s a bit of a paradox at the moment that it’s disorganized, research is of low quality, but the potential is fantastic,” according to an opinion leader in the use of eHealth technology for health care delivery systems. Going forward, several stakeholders recommended that, rather than focus on the development of new applications, the field of eHealth should concentrate on the stability, usability, and applicability of technologies within the existing infrastructure.

### Theme 4: Health Disparities–eHealth as a Bridge or Another Hurdle?

There was a range of opinions about the ability of eHealth to reach populations without access to routine, traditional care. Stakeholders with a macro- or policy-oriented perspective (developers, researchers, IT opinion leaders) were confident that technology has the ability to surmount factors (e.g., reading literacy, distance and time constraints, language fluency) that contribute to limited access to health care. Additionally, eHealth applications have the ability to be tailored according to users’ attributes. Race/ethnicity, age, and gender were often-cited dimensions, but more nuanced attributes (e.g., cultural and linguistic diversity within ethnic groups, or socioeconomic status) were not detailed in discussions about tailoring.

It was argued that technology, particularly computers, will become increasingly ubiquitous and affordable, as occurred with the widespread adoption of televisions and telephones in the 20th century. In some research studies, individuals from traditionally underserved populations have shown greater improvements in outcomes and higher degrees of interest than middle-class subjects [30].

Stakeholder within a National Physician Organization [T]his is just an opinion, I can’t defend this with data yet, but it just seems to me that increasingly the question is about rich and poor, regardless of ethnicity and regardless of race…. My own personal view of e-technologies and eHealth is that at some fundamental societal level, this is an act of democratization of information and that, overall, it’s just incomprehensible to me that this would not help disadvantaged populations more than it helps advantaged populations…. It’s almost certainly going to be a help, not a hindrance.

eHealth IT Opinion Leader All of us…have a huge responsibility and challenge in front of us to try to get people to think that eHealth is not a computer and a broadband connection and you’ve got to have wireless, and you’ve got to have this or that. In fact, it’s just the opposite…. It’s an enabling technology.

Other participants, particularly those with day-to-day interactions with patients and health systems, had a more immediate, pragmatic set of concerns. While they did not discount the potential for well-designed technology to impact risk behaviors in individuals, they were skeptical that those who are traditionally underserved by the health care system will ever truly have equal access to state-of-the-art technology, and they are concerned about a disparity in technological literacy and aptitude:

Member of a Physician Organization/Provider Group I think in its current state, [eHealth] is probably widening the gap. I think that it has the potential to narrow the gap, but I don’t think people have focused on that sufficiently…. I don’t think that we are lessening this divide because the underserved are also underserved as far as their access and time to go on the Web.

Most stakeholders agreed that, once the issue of access to technology is resolved, eHealth has a great deal of promise for addressing health disparities. The operationalizability of the tools was not nearly as much a concern as access, because programs can be designed to accommodate low technological literacy and because underserved populations have demonstrated a high degree of interest in these technologies. The hotly debated topic was whether it was more cost-effective to invest limited health care dollars in eHealth as a means of reaching these populations, or to instead channel financial resources into publicly financed insurance systems like Medicaid. It was recommended that more research, especially qualitative studies, be dedicated to understanding exactly what underserved populations need in terms of access to health care and technology, as well as utilization abilities and patterns once access is achieved.

## Discussion

### Comparative State of eHealth

These stakeholder interviews covered many of the topic areas that have been outlined in the eHealth literature, including the need for an evidence base in eHealth and methodological issues associated with research in this field, challenges for implementation, and emerging trends and future directions [24]. While similar in scope, the emphasis of these interviews differs from previous stakeholder research conducted in the United Kingdom, which used similar methods and lines of inquiry. Jones et al [24] interviewed professionals with a high level of interest in eHealth (i.e., health care providers, academic eHealth researchers, developers), as well as policy makers. That study found that stakeholders called foremost for research on eHealth to demonstrate any cost-effectiveness and evidence of improved quality of life. Second on the list of concerns were topics related to the control and transmission of information (i.e, confidentiality and security). In contrast, the stakeholders in the United States spent more time discussing specific methodological challenges to development, research, and evaluation. While they overlapped with the stakeholders in the United Kingdom in their lengthy discussions of human factors and behavioral research to inform a larger eHealth research paradigm, US stakeholders gave slightly higher priority to evaluating existing technologies and applications. Stakeholders in both countries were mindful of the need for demonstrated effects on health outcomes. Notably, the role of eHealth in addressing disparities in access to health care achieved greater prominence in the United States than in the United Kingdom. This is likely due to differences in the administration of health care between the two countries: the United Kingdom’s nationalized system, which is essentially free at point of service, creates a different set of circumstances compared to the mainly private, insurance-based health care model in the United States. This key difference could explain incongruent findings on other points as well: the stakeholders in the United States may find it more compelling to first address user-oriented concerns (i.e., defining measures for individually oriented applications, developing patient-centered technologies that address prevention and disease management to aid time- and resource-strapped physicians in patient care), while stakeholders in the United Kingdom might find more benefit in ensuring systemic integrity and carefully evaluating whether eHealth applications help citizens to achieve greater quality of life in a way that is cost-effective to the nationalized system.

It is also possible that these differences are due to the several limitations that must be considered in the interpretation and presentation of these findings. First, the sample was not representative of the defined stakeholder categories due to the nonrandom selection of participants. Second, there were not an equal number of stakeholders in each category, although similar proportions were represented in this sample as in the United Kingdom stakeholder study discussed above. Perhaps most importantly, the focus of the interviews was skewed towards the interviewers’ aims of informing a research agenda and funding priorities. The line of inquiry included several questions on evaluation methods, as the participants were chosen from a list of collaborators on an initiative that stressed the importance of evaluation. A larger, random sample with more candid, participant-led conversation may have elicited different opinions or levels of interest in evaluation and research methodology.

The primary goal of the stakeholder interviews, namely to establish a research agenda and funding priorities for the Health e-Technologies Initiative, was achieved successfully. Subsequent to these interviews, the Health e-Technologies Initiative funded 24 grants over the following two years, addressing many of the issues identified through the interview process. Descriptions of grantee research projects are maintained on the Initiative’s website (http://www.hetinitative.org). The results of these conversations were also helpful as the Initiative sought to promote evaluation standards and multidisciplinary collaboration among researchers. In addition, it has been valuable to examine these research findings on an aggregate, retrospective level in order to assess the progress that has been made in the field since 2002/03 toward building eHealth’s credibility and future potential.

Clearly, advancements have been made. A recent study noted an 84% rise in the publication of articles from the period 1995–99 to 2000–04 that included the term *behavioral informatics,* one of the many phrases sometimes used interchangeably with *eHealth* [31]. As mentioned above, work continues on defining and clarifying the meaning of *eHealth,* not only to enhance communication between those who interact within this discipline [25], but also “to identify its place within the wider health informatics field, as part of a larger review of research and expert analysis pertaining to current evidence, best practice and future trends” [26].

Other markers point to expanding interest in eHealth evaluation research. The Health e-Technologies Initiative was the first national program of The Robert Wood Johnson Foundation with eHealth research as a core focus. The Initiative’s 2002 call for proposals, designed to solicit a broad range of project ideas, generated 600 letters of intent. Even the more narrowly focused 2004 call for proposals drew 99 first-round applicants. In June of 2005, the National Cancer Institute, the National Institute of Mental Health, the National Institute on Drug Abuse, the National Library of Medicine, the Office of Behavioral and Social Science Research, the Office of Disease Prevention/NIH, and the Health e-Technologies Initiative sponsored the first Critical Issues in eHealth Research Conference, which was attended by 400 participants from across North America.

As eHealth continues to be defined and its value and limitations are demonstrated, it will become increasingly important to standardize evaluation approaches and promote collaboration among sectors in order to achieve optimal dissemination and cost-effective, population-level improvements in health outcomes. While these interviews showed some degree of satisfaction and consensus in various realms within eHealth evaluation research, the stakeholders’ collective insights and thoughts highlight the relative nascent stage of this work, and offer guidance as to areas of future exploration, which are outlined below.

### Research and Policy Recommendations

The proposed research and policy recommendations for eHealth are summarized in the Textbox. An evidence-based approach is key to achieving eHealth’s future potential. In order to establish cohesive, standardized process and outcome measures, rigorous evaluation efforts must be made across both public and private sectors. Evaluation results should be widely disseminated to developers in order to establish industry standards. The uptake of eHealth by purchasers, as well as consumers, will be more likely if formal standards of quality and effectiveness are available to assist in informed decision making about available eHealth applications. In order to address concerns about users accessing misguided, erroneous, or inappropriate health information on the Internet, it is important to continue to define measures of quality and perceived credibility. These measures will inform the development of formal standards and accreditation mechanisms for IHCs, allow researchers to demonstrate the prevalence and risk presented by inaccurate websites, and provide guidance to practitioners and users as they navigate the Internet for health resources [32–35]. The emergence of convincing evidence of the effectiveness of eHealth programs will enable policy makers to include eHealth in ongoing efforts to refocus national programs such as Medicare and Medicaid on prevention and chronic disease management.

eHealth has the capacity to address health disparities among traditionally underserved populations due to its scalability, potential to target specific groups and conditions, and ability to be tailored and customized to culturally and linguistically diverse users [36,37]. It is strongly recommended that qualitative research and field trials be performed to understand the preferences and technological needs of underserved populations. While technology platforms that support eHealth are likely to become ubiquitous in the future, special attention should be paid to incorporating technology into environments where the underserved may access these services. Reducing health disparities is a major objective of Healthy People 2010 [38], and eHealth has the potential to help the nation achieve that policy imperative.

Inevitably, the infrastructure of health systems must be considered in efforts to broadly disseminate eHealth applications. The enthusiasm with which health care providers incorporate eHealth into routine care is contingent upon how well technologies are integrated into the workflow of health care environments where work is too often constrained by reimbursement structures and lack of time [39,40]. In addition to human factors, technological interoperability must be ensured in order to facilitate the widespread use of eHealth across health systems and among physicians, users, and administrators [39,41]. Reimbursement incentives are also important to consider when proposing eHealth solutions that supplement or replace standard care, as it is important to engage physicians in eHealth dissemination efforts. Opportunities exist in eHealth to link disparate members of the health care system with patients and their proxies in new ways, in order to achieve more consistent care [42]. These linkages may ultimately result in better patient health outcomes, which is an area that warrants further investigation when researching the efficacy and cost-effectiveness of eHealth applications [24].

**Research and policy recommendations**

**Recommended Areas for Future Research:**

- Refinement of process and outcome measures
- Enhancement of sampling and recruitment strategies
- Efficacy of individual tailoring
- Effectiveness of targeting high-risk populations
- Long-term cohort studies on cost-effectiveness
- Appropriate use of technology for integrating health care
- Incentives for health care providers to incorporate eHealth applications into routine care
- Qualitative studies on the health and technology-related needs and preferences of underserved populations

**Recommended Policy Priorities:**

- Establish accreditation mechanisms to standardize, approve, and monitor the development of quality eHealth applications.
- Incorporate emerging technologies into environments occupied by traditionally underserved groups.
- Foster technological interoperability to promote eHealth connectivity.
- Implement evidence-based eHealth solutions to transform and enhance health care provision.
